# Eccrine malignancies in nevus sebaceus: Case presentation and review of the literature

**DOI:** 10.1016/j.jdcr.2024.12.029

**Published:** 2025-01-13

**Authors:** Alexander D. Woods, Wenhua Liu, Aleksandar L. Krunic

**Affiliations:** a Department of Dermatology, University of Illinois Chicago College of Medicine, Chicago, Illinois; b Department of Dermatology, Cook County Health, Chicago, Illinois; c Department of Dermatology, Northwestern University Feinberg School of Medicine, Chicago, Illinois

**Keywords:** dermatologic surgery, eccrine porocarcinoma, nevus sebaceus of Jadassohn, RAS genes, slow Mohs surgery, sweat gland neoplasms

## Introduction

Nevus sebaceus (NS), or nevus sebaceus of Jadassohn, is a benign hamartomatous tumor usually present at birth as a hairless flat to minimally raised plaque.^1^ NS typically grows proportionally with the body until puberty, when it becomes more prominent due to the hormonal effects of androgens on sebaceous glands.^2^ Patients often present to dermatology clinics at this time, when the tumor is more pronounced, verrucous, and yellowish-orange.

While NS are benign tumors, they have a high propensity to develop secondary neoplasms, especially in lesions of the scalp, stemming from underlying mosaic postzygotic HRAS and KRAS gene mutations.^1^^,^^3^ These neoplasms are predominantly benign; however, rarely, secondary malignant tumors may arise, and exceptionally sweat gland carcinomas.^1^^,^^4^, ^5^, ^6^, ^7^, ^8^

Herein, we present a case of porocarcinoma and syringocystadenoma papilliferum (SCAP) collision tumors arising in the background of a NS.

## Case report

**Fig 1:**
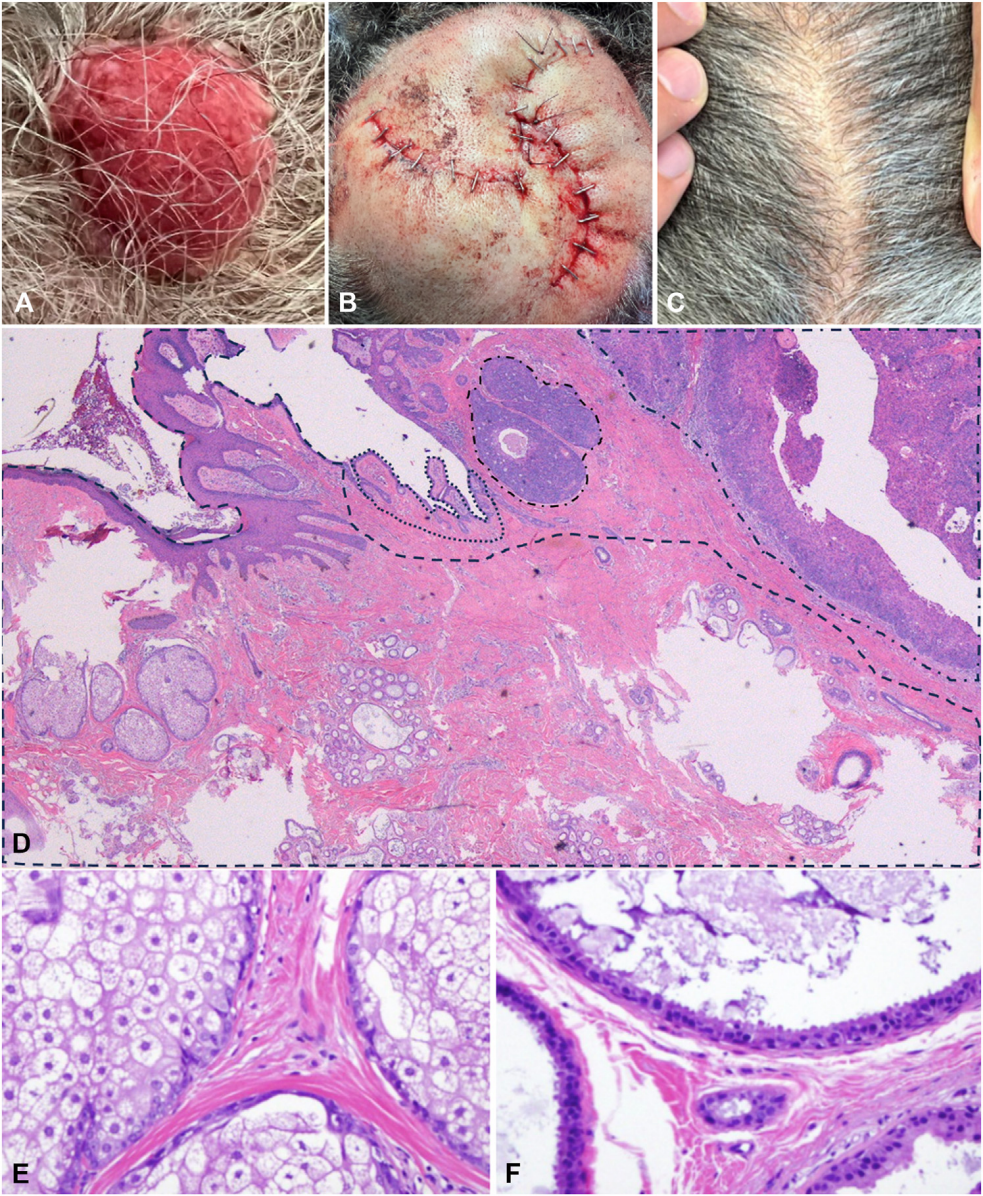
**A,** Friable, *red*, dome-shaped nodule on the medial parietal scalp measuring 3 cm in diameter; (**B**) repaired with O-Z double rotational flap; (**C**) follow-up at 24 months without recurrence; (**D**) All 3 neoplasms at low power (hematoxylin and eosin [H&E] 10×); left to right: (*dashed line*) nevus sebaceus with abortive hair follicle, sebaceous glands, numerous apocrine ducts and glands; (*dotted line*) syringocystadenoma papilliferum; (*mixed line*) nodules of porocarcinoma; (**E**) higher magnification of sebaceous glands (H&E 200×); (**F**) higher magnification of apocrine glands with decapitation secretion in the remaining part of the nevus sebaceus (H&E 400×).

The 77-year-old African American female patient presented with the aggressive development of a nodule on a pre-existing NS at the mid vertex of her scalp. The 3 × 3 cm red, friable, dome-shaped nodule had reportedly tripled in size over the last 6 months (Fig 1, *A*). Due to the rapidly progressive nature, size, and risk of missing individual components of the suspected collision tumor, the decision was made to proceed with staged excision with 10 mm margins and “en face processing” (slow Mohs surgery). Once clear margins were confirmed, the defect was repaired with an O-Z double rotational flap (Fig 1, *B*).

**Fig 2:**
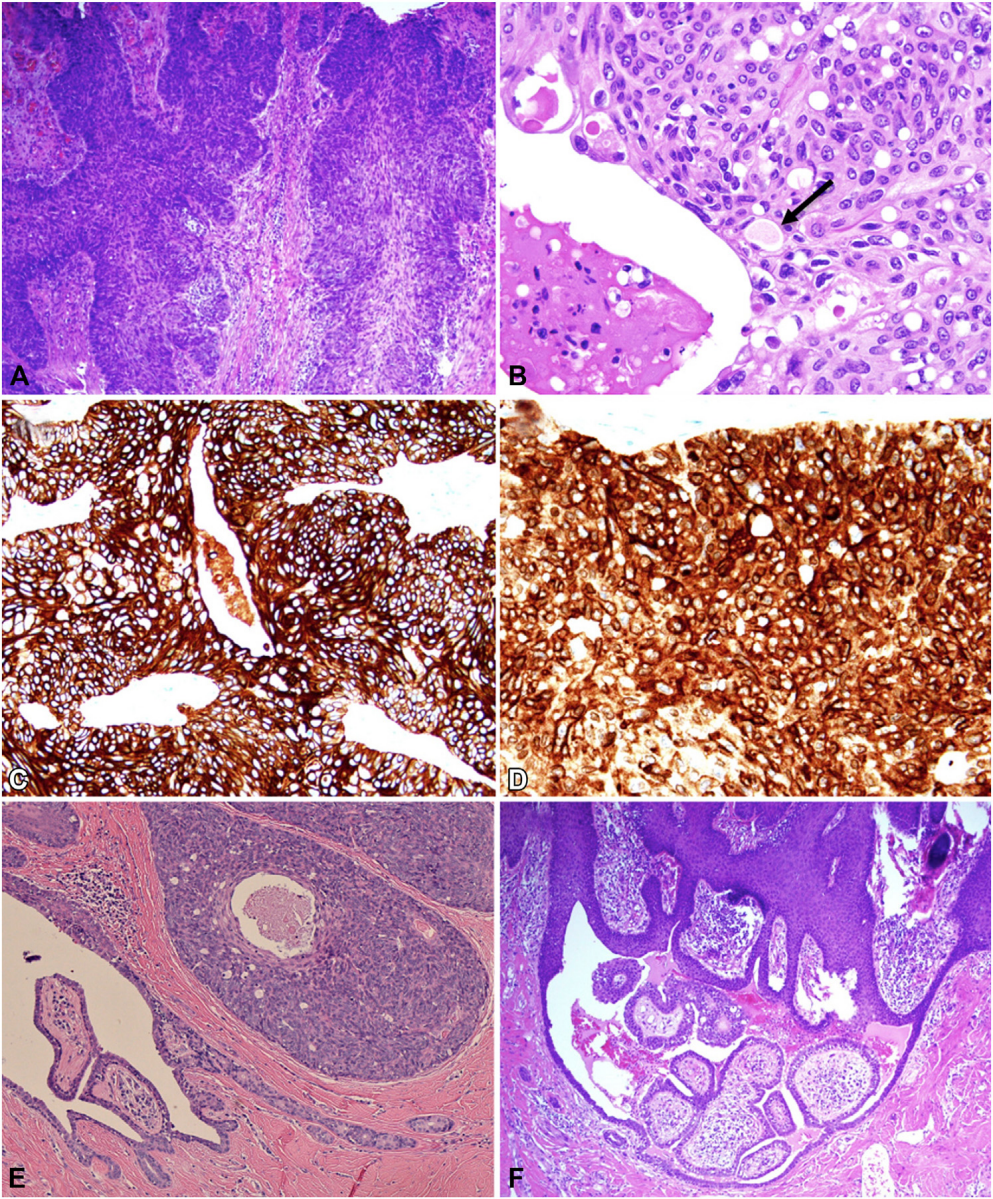
**A,** Porocarcinoma proliferation of severely atypical epithelial cells in the epidermis with infiltrative extension into the dermis (hematoxylin and eosin [H&E] 100×); (**B**) higher magnification of porocarcinoma with evidence of microduct formation (*black arrow*), multiple mitotic figures, and cystic degeneration with necrotic debris (H&E 400×); (**C**) Diffuse strong cytokeratin 7 (CK7) staining of neoplastic cells (200×); (**D**) Diffuse strong epithelial membrane antigen (EMA) staining of neoplastic cells (200×); (**E**) higher magnification of syringocystadenoma papilliferum and adjacent porocarcinoma with evidence of comedo necrosis and microducts (H&E 40×); (**F**) different plane of syringocystadenoma papilliferum with typical papillary projections and associated cystic spaces (H&E 40×).

Histopathologic examination with hematoxylin and eosin (H&E) revealed a proliferation of severely atypical epithelial cells in the epidermis with infiltration into the deep dermis adjacent to areas of focal ulcer (Figs 1, *D* and 2, *A*). There was prominent nuclear pleomorphism, eosinophilic-to-clear cytoplasm, frequent mitotic figures, and focal microduct formation (Fig 2, *A* and *B*). The tumor cells stained strongly positive for cytokeratin 7 (CK7) (Fig 2, *C*) and epithelial membrane antigen (EMA) (Fig 2, *D*), and negative for androgen receptors, favoring eccrine porocarcinoma over sebaceous carcinoma. Adjacent sections revealed epidermal hyperplasia, papillomatosis, and orthokeratotic hyperkeratosis, with aggregates of sebaceus glands located abnormally high in the dermis and prominent apocrine glands, consistent with NS (Fig 1, *D*-*F*). Furthermore, there were cystic invaginations of infundibular epithelium extending from the epidermis into the dermis, lined by 2 cell layers of columnar and cuboidal cells, with stroma rich in plasma cells, consistent with SCAP (Figs 1, *D* and 2, *E* and *F*). The diagnosis was made of collision tumors of porocarcinoma and SCAP in a NS.

Tumor tissue was sent for molecular testing and was positive for pathogenic HRAS G13R missense mutation, c.37G > C (p.G13R) in one-third of the tissue. In addition, TP53 mutation in exon 6, c.638G > T (p.R213L), and loss of retinoblastoma protein (Rb) staining in 95% of tissue were revealed. Furthermore, there was diffuse loss of p16 expression in porocarcinoma cells.

Computed tomography of the head and neck was performed and did not show any metastases. Follow-up through 2 years has been unremarkable (Fig 1, *C*).

## Discussion

Porocarcinoma, or eccrine porocarcinoma, is a rare, aggressive sweat gland neoplasm that is thought to arise from the acrosyringium, the intraepidermal spiral duct of the eccrine apparatus.^9^ It most commonly appears in the seventh-eighth decade of life on the head/neck or legs, without consistent sex or ethnicity predilection. Porocarcinomas may arise de novo or as a malignant transformation of benign cutaneous lesions, most commonly eccrine poromas (18% to 50%).^9^^,^^10^ Exceptionally, porocarcinomas have been described as occurring in NS, with only 2 cases reported in the literature.^6^^,^^7^ Both tumors were located on the scalp, and one of the cases presented as a collision tumor with a trichoblastoma.^6^^,^^7^

NS, though a benign hamartoma, has a high propensity for the development of secondary neoplasia (10% to 22%), often in the fourth decade and beyond. These are most commonly benign neoplasms, like trichoblastoma (∼5.0% of NS) or SCAP (∼4.3% of NS).^1^^,^^4^^,^^5^ Single lesions of NS can sometimes harbor 2 (∼2 to 4% of cases), rarely 3, or even 8 different neoplasms as collision tumors.^1^^,^^2^^,^^4^ Secondary malignant neoplasms are reported in 0.8% to 2.5% of NS.^1^^,^^4^ While the most common malignancy reported is basal cell carcinoma, other aggressive tumors have been described, like squamous cell carcinoma, sebaceous carcinoma, and even melanoma.^1^^,^^4^^,^^5^ Sweat gland tumors like apocrine carcinoma, microcystic adnexal carcinoma, poromas, and porocarcinomas have also been exceptionally rarely reported in NS (Table I).^1^^,^^4^^,^^8^^,^^11^^,^^12^ Most of these secondary tumors were on the scalp, emphasizing the importance of this localization for monitoring and aggressive treatment of any NS lesion that demonstrates sudden change in appearance, especially after the third or fourth decade of life. Hence, some authors recommend immediate complete excision of such lesions even without a previous biopsy.^5^ Performing partial (punch or shave) biopsy before excision in these lesions carries a risk of incomplete histopathological assessment and missing a malignant component, as there may be several tumors present.

**Table I:** Rare sweat gland neoplasms of nevus sebaceus

Tumor type	Number	Age range (mean), y	Male:female	Locations	Association with another secondary neoplasm
Poroma∗	7	11-63, (36)	2:4	Scalp ×5, cheek ×1	4/6
Porocarcinoma	3	45-77, (60)	1:2	Scalp	2/3
Apocrine carcinoma∗	13	45-77, (65)	7:4	Scalp	3/11
Microcystic adnexal carcinoma∗	5	16-75, (49)	3:1	Scalp ×3, cheek ×1	1/4

∗ Specific data unable to be obtained on all patients.

Recent genetic analyses demonstrated that up to 95% of NS are associated with a postzygotic mosaic mutation in the HRAS system, namely c.37G > C (p.G13R). This HRAS mutation leads to constitutive activation of MAPK and PI3K-Akt signaling pathways and increased cellular proliferation.^3^ This mutation is also detected in associated secondary neoplasms like SCAP and trichoblastoma, and even in some cases of eccrine poroma, regardless of association with NS.^3^^,^^13^ Minowa et al described an additional mutation of the TP53 system (c.473G > C) in a coexisting eccrine poroma in their case of NS.^14^ Both mutations may significantly disinhibit the already proliferative environment from activating mutations in the HRAS system, which works as a GTPase and induces cell growth, proliferation, differentiation, and survival.^3^^,^^15^ Rajasekharan et al explain the role of an intact TP53 system inducing alternate splicing of mutated HRAS genes, providing measures to eliminate oncogenic mutated HRAS-induced neoplastic proliferation.^15^ Subsequent loss of the TP53 system could therefore contribute to the development of an eccrine poroma component in a NS. Further regression/malignant transformation of an eccrine poroma may require an additional mutation in the tumor suppressor Rb gene, which could be a plausible mechanism of the development of porocarcinoma in collision with SCAP in an NS, as demonstrated in our case.

Early detection of porocarcinoma and other aggressive sweat gland neoplasia in a NS is paramount. Porocarcinoma is an aggressive neoplasm that may grow rapidly over weeks to months.^9^ Treatment of primary lesions is with surgical excision, classically wide local excision; however, Mohs micrographic surgery has been increasingly utilized, especially in tumors with high-risk features, with reports of significantly decreased recurrence and metastasis rates.^10^ These features include tumor depth greater than 7 mm, lymphovascular invasion, greater than 14 mitoses per high-power field, and an “infiltrative” lower margin.^9^

Prognosis is good in early-stage porocarcinomas; however, local recurrence rates have been reported at around 20% with surgical excision alone.^9^ Furthermore, metastatic disease has been reported at presentation in up to 31% of cases, most commonly regional lymph nodes, although this is site-dependent, with porocarcinomas of the head and neck being least associated with metastatic disease.^9^^,^^10^ The presence of lymph node metastasis has shown mortality rates of 67%, with a 3-year survival of 39.5%.^9^ Early recognition and treatment of porocarcinoma is paramount to prevent disease-related mortality, and the rare presentation of this case highlights the need to monitor NS closely for any morphologic changes postpuberty. Our patient has done well, with no signs of tumor recurrence after 2 years of monitoring and follow-up imaging.

## Conclusion

Porocarcinoma has rarely been described to arise in NS, with only 2 previous cases reported. This case highlights the propensity of NS for multiple neoplastic proliferations initiated by the proliferative effects of constitutively activated HRAS by mosaic postzygotic gene mutations. Subsequent mutations in genes like TP53 and Rb can lead to the development of secondary neoplasia, some of which can be aggressive, necessitating complete excision, especially for lesions on the scalp. Sweat gland tumors are rare in the scope of NS, but when present, necessitate immediate complete removal. Partial biopsies are not recommended due to the risk of missing individual tumors, which may present in collision in the settings of NS.

## Conflicts of interest

None disclosed.
